# FAR-Net: Feature-Wise Attention-Based Relation Network for Multilabel Jujube Defect Classification

**DOI:** 10.3390/s21020392

**Published:** 2021-01-08

**Authors:** Xiaohang Xu, Hong Zheng, Changhui You, Zhongyuan Guo, Xiongbin Wu

**Affiliations:** 1 School of Electronic Information, Wuhan University, Wuhan 430072, China; xuxiaohang@whu.edu.cn (X.X.); youchanghui@whu.edu.cn (C.Y.); guozhongyuan@whu.edu.cn (Z.G.); xbwu@whu.edu.cn (X.W.); 2 School of Cyber Science and Engineering, Wuhan University, Wuhan 430072, China

**Keywords:** attention mechanism, relation network, jujube defect inspection, multilabel classification

## Abstract

In production, due to natural conditions or process peculiarities, a single product often may exhibit more than one type of defect. The accurate identification of all defects has an important guiding significance and practical value to improve the planting and production processes. Concerning the surface defect classification task, convolutional neural networks can be implemented as a powerful instrument. However, a typical convolutional neural network tends to consider an image as an inseparable entity and a single instance when extracting features; moreover, it may overlook semantic correlations between different labels. To address these limitations, in the present paper, we proposed a feature-wise attention-based relation network (FAR-Net) for multilabel jujube defect classification. The network included four different modules designed for (1) image feature extraction, (2) label-wise feature aggregation, (3) feature activation and deactivation, and (4) correlation learning among labels. To evaluate the proposed method, a unique multilabel jujube defect dataset was constructed as a benchmark for the multilabel classification task of the jujube defect images. The results of experiments show that owing to the relation learning mechanism, the average precision of the three main composite defects in the dataset increases by 5.77%, 4.07%, and 3.50%, respectively, compared to the backbone of our network, namely Inception v3, which indicated that the proposed FAR-Net effectively facilitated the learning of correlation between labels and eventually, improved the multilabel classification accuracy.

## 1. Introduction

### 1.1. Multilabel Jujube Defect Classification

**Figure 1:**
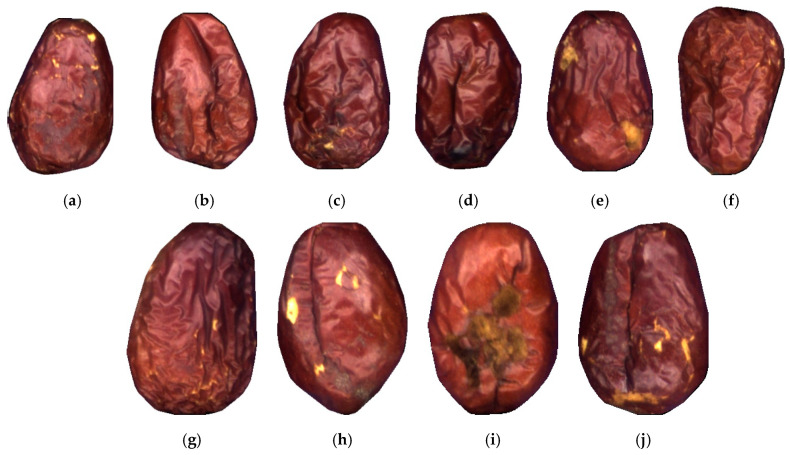
Examples of multilabel jujube defect samples: (**a**) russeting + peeling; (**b**) russeting + cracking; (**c**) mild rot + peeling; (**d**) mild rot + cracking; (**e**) bird pecking + peeling; (**f**) shriveled + peeling; (**g**) russeting + peeling + mild rot; (**h**) russeting + peeling + cracking; (**i**) mild rot + cracking + peeling; (**j**) bird pecking + severe rot + cracking.

Generally, in the surface defect classification task, samples and labels are in one-to-one correspondence. That is, a sample usually contains only one type of a defect feature, which is referred to as the single-label classification problem. However, in actual production, there may be more than one kind of defect in a single product. Figure 1 represents several samples in the multilabel dried jujube defect dataset considered in the present research. It can be seen that each sample contains at least two different defects. Among them, *peeling* and *cracking* are the two most common types of defects in jujube products, while *mild rot*, *severe rot*, and *bird pecking* are always accompanied by *cracking* symptoms according to a priori knowledge. Exploring and learning internal connections between different labels is of great importance in improving the classification accuracy of multilabel samples. Therefore, developing an appropriate classification method for the multilabel jujube defect has the considerable practical value for production and research.

In recent years, deep models based on convolutional neural networks (CNN) have demonstrated superior performance in various image classification tasks, such as target recognition and detection. The intensive matrix calculation and rich perception ability of CNNs are particularly suitable for feature extraction and mapping. However, at present, the direct application of CNN to the multilabel defect classification task still provides unsatisfactory results due to following reasons: (1) A typical CNN tends to consider an image as an inseparable entity and a single instance when extracting features. If different label features are combined in a single instance, it is difficult to discriminate between them. (2) As CNN does not have an appropriate expression mechanism for semantic relations and dependencies among labels, label correlations are often overlooked.

Therefore, in the present research, we aimed to investigate the ways to exploit the advantages of feature expression in deep learning, to enhance the learning of label correlations, and to improve the accuracy of multilabel classification.

### 1.2. Review of the Deep Learning-Based Method for Multilabel Classification

In recent years, to address a series of challenges in the application of deep learning to multilabel classification, researchers have introduced various models and architectures, and some of them have achieved notable results. These methods mainly include the approaches described below in detail.

#### 1.2.1. CNN-Based Methods

Although the original CNN model is not suitable for direct application to the multilabel classification problems, it can still be used to achieve better performance by improving the loss function or classifier. L. Zhang et al. [1] proposed a multitask CNN model that formulated each label learning as a binary classification task and transformed multilabel learning into the multiple binary classification tasks by improving the loss function. Y. Liu [2] proposed a multilabel image classification model based on deep metric learning that combined deep neural networks with discriminative metric learning. It retained the discriminate information of a sample while learning nonlinear mapping and achieved better classification accuracy.

Furthermore, Y. Gong et al. [3] developed a CNN-based model combined with the weighted approximate-rank pairwise (WARP) loss function to complete the multilabel classification task and analyzed in detail several key elements that had a direct impact on improving accuracy. The model sorted the prediction results and then used K results with the largest confidence as prediction labels. In addition, Y. Wei et al. [4] introduced the hypotheses CNN pooling (HCP) algorithm that implied dividing an input image into different small patches, then inputting each patch to the same CNN, and finally, implementing the max pooling layer to predict the results for all patches. The results were aggregated to produce the final multilabel result. It can be seen that although the WARP and HCP algorithms achieved acceptable classification performance on several multilabel benchmark datasets, neither of them incorporated correlation learning among labels.

#### 1.2.2. RNN-Based Methods

As an alternative to the CNN-based methods, several researchers proposed to apply a recurrent neural network (RNN) to learn semantic connections between labels. The input and output of a traditional neural network can be considered relatively independent. In RNN, however, each output is associated with previous multiple inputs. This structure provides RNN with the ability to remember and capture long-term dependent information.

**Figure 2:**
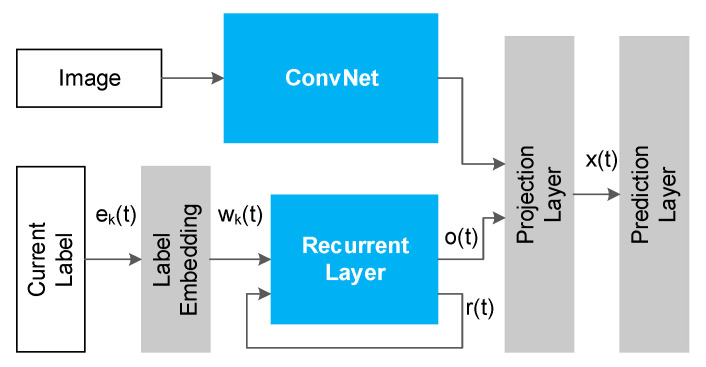
Architecture of the convolutional neural networks (CNN)-recurrent neural network (RNN) model.

J. Wang et al. [5] introduced a CNN-RNN model to realize multilabel image classification. The method comprised two parts: the CNN module was responsible for image feature extraction and the RNN module was designed to model the relationship between an image and a label, as shown in Figure 2. CNN-RNN realized correlation learning through mapping the image and label features into the same lower dimensional space. This method transformed the multilabel classification problem into the label-prediction sequence problem. For example, concerning the labels “Sky” and “Airplane”, there were two predicted paths (“Sky”, “Airplane”) and (“Airplane”, “Sky”). The probability of each path was calculated by RNN. While training the CNN-RNN model, it was necessary to manually set the order of label prediction.

Several other notable RNN-based approaches include regional latent semantic dependencies model (RLSD) [6], recurrent memorized attention model (RMA) [7], and recurrent attention reinforcement learning model (RARL) [8]. Among them, RLSD and RMA are relatively similar. They both use CNN to extract image features, and then apply RNN or long short-term memory (LSTM) [9] to learn the position of a label in a feature map to enhance the feature response corresponding to the position. Finally, the enhanced feature is employed to predict the label. RARL applies reinforcement learning to construct semantic connections between labels.

Analysis of the aforementioned methods has indicated that although they have achieved a significant improvement in terms of classification accuracy, it is difficult for them to accurately and completely predict all labels that may exist due to the uncertainty in the number of labels in an input image. In addition, the RNN-based methods usually are associated with high computational costs and large memory requirements, which is not applicable to the application of the defect inspection models in actual production.

#### 1.2.3. Attention-Based Methods

The attention mechanism (AM) was introduced in the field of image processing in the early 1990s. Its essence is grounded on the human visual attention system, that is, when human vision perceives something, it usually does not see the entire scene but observes and pays attention to specific parts according to needs. Furthermore, when humans realize that a target to observe often appears in a certain area or location of a scene, they learn subconsciously and focus on that particular area when similar scenes appear. In 2014, V. Mnih et al. [10] induced AM to become a widely researched topic in deep learning. In their research work, an RNN-based model combined with AM was applied to the image classification tasks. After that, D. Bahdanau et al. [11] applied AM to natural language processing, aiming to achieve simultaneous translation and alignment in the machine translation tasks. In 2017, A. Vaswani et al. [12] utilized the self-attention method to learn the representation of textual features. At present, AM has been widely used in the field of image processing, including classification, detection, and other tasks, and has achieved encouraging results.

**Figure 3:**
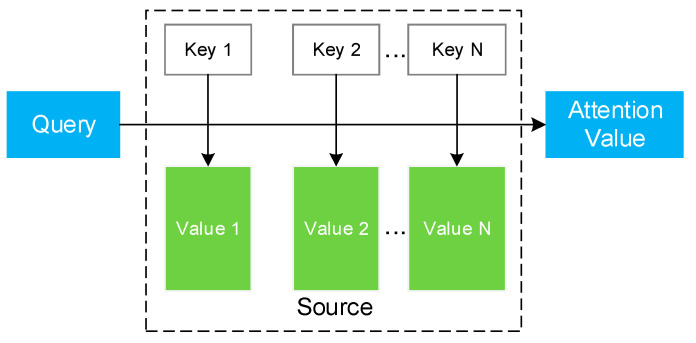
Schematic diagram of the attention algorithm.

As shown in Figure 3, the attention algorithm can essentially be described as a query mapping to a series of key-value pairs, similarly to the addressing process:

(1) $$Attention\left(Query,Source\right)=\sum_{i=1}Similarity\left(Query,Key_{i}\right)\cdot Value_{i}$$

Specifically, the estimation of attention can be divided into the following three steps:

- Calculate the similarity between query and each key to obtain the corresponding weight. Commonly used similarity algorithms include the dot product:
  (2) $$Similarity\left(Query,Key_{i}\right)=Query\cdot Key_{i}$$
  cosine similarity:
  (3) $$Similarity\left(Query,Key_{i}\right)=\frac{Query\cdot Key_{i}}{\left\|Query\|\cdot \|Key_{i}\right\|}$$
  multi-layer perceptron (MLP):
  (4) $$Similarity\left(Query,Key_{i}\right)=MLP\left(Query,Key_{i}\right)$$
  and concatenation, etc.
- Use Softmax or other functions with similar characteristics to normalize all weights:
  (5) $$a_{i}=Softmax\left(Sim_{i}\right)=\frac{exp\left(Sim_{i}\right)}{\sum_{j=1}exp\left(Sim_{i}\right)}.$$
- The weight and the corresponding value are weighted and summed to obtain the final attention value:
  (6) $$Attention\left(Query,Source\right)=\sum_{i=1}a_{i}\cdot Value_{i}.$$

There are several noteworthy works focused on the AM-based multilabel classification methods. B. Wei et al. [13] proposed a bio-inspired visual integrated model (BIVI-ML) for multilabel textile defect classification. In BIVI-ML, three bio-inspired visual mechanisms (the visual gain, visual attention, and the visual memory ones) were constructed to improve resolution and feature discrimination, identify textile defects, and associate relevant labels, respectively. Z. Yan et al. [14] introduced a feature attention network (FAN) to implement multilabel classification that included the feature refinement and correlation learning networks. FAN established a top-down feature fusion mechanism to refine more important features and learn label dependencies. Y. Hua et al. [15] proposed a novel end-to-end network, namely class-wise attention-based convolutional and bidirectional LSTM network (CA-Conv-BiLSTM), for multilabel aerial image classification. The network comprised three key components: a feature extraction module, a class attention learning layer, and a bidirectional LSTM-based subnetwork. The above BIVI-ML and CA-Conv-BiLSTM models both used LSTM for label association learning, which required complex calculations and had unsatisfying inference efficiency. The FAN model was mainly aimed at small targets and tail labels in a rather large dataset, which was unsuitable for multilabel jujube defect classification.

In summary, the deep learning-based multilabel image classification method has advanced considerably in recent years. However, there are still deficiencies that have not been solved perfectly. In this regard, in the present study, we further explored the construction of deep networks for multilabel jujube defect classification.

## 2. Feature-Wise Attention-Based Relation Network

According to the previously discussed methods, it is extremely important to strengthen the label correlation learning of a deep learning network to better solve the multilabel jujube defect classification problem. This is because the certain types of defects often appear in pairs due to material properties or environmental reasons. Therefore, an effective deep learning network should have the following capabilities:

(1) Reliable feature extraction. Feature extraction is the most preconditioned part in a machine vision system. Specifically in multilabel classification, the information contained in a multilabel sample is more abundant than that in a single-label sample. Therefore, a reliable feature extraction module is required to ensure that the effective knowledge about a sample is extracted completely and learned;
(2) Label-wise feature aggregation. After obtaining the overall valid feature information about a multilabel sample, the label-wise aggregation of feature maps is required to learn dependencies and connections between different labels in subsequent modules.
(3) Activation and deactivation of label features. As a single sample usually does not contain all kinds of defects, it is necessary to further filter the aggregated label features. That is, the feature maps corresponding to the labels that do not exist in a sample are required to be deactivated, and the remaining label features need to remain activated.
(4) Comprehensive learning of the correlation among label features. Undoubtedly, this is the most conclusive module in a multilabel classification network. Whether a semantic relation between different defects can be completely learned, it determines the multilabel classification performance of a network.

**Figure 4:**
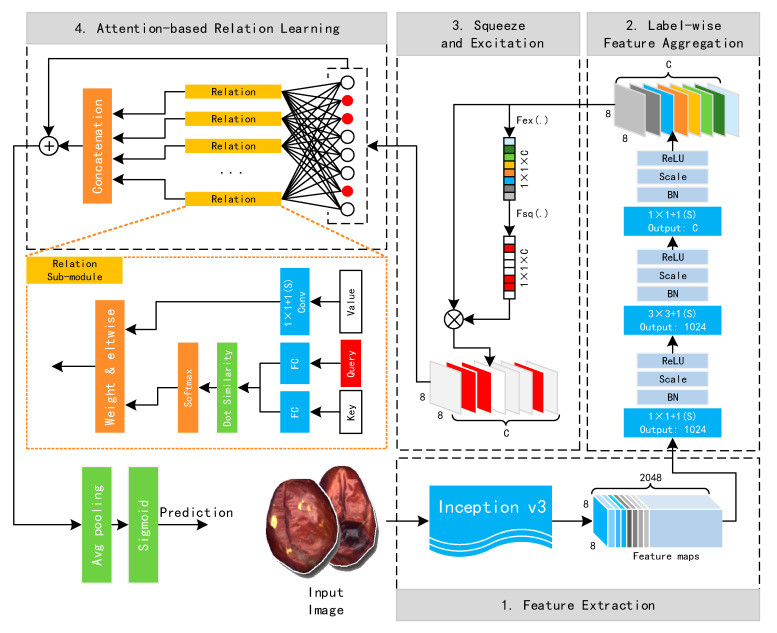
Structure of the feature-wise attention-based relation network (FAR-Net).

Based on these considerations, we propose a feature-wise attention-based relation network (FAR-Net) for multilabel jujube defect classification. FAR-Net includes four different modules: feature extraction (FE), label-wise feature aggregation (LFA), activation and deactivation (ADA), and attention-based relation learning (ARL). The overall structure is represented in Figure 4. The four modules are clearly divided and depicted in the figure. Next, we further elaborate and explain the details and mechanisms of these four modules.

### 2.1. Feature Extraction

The feature extraction network is the first consideration of FAR-Net and serves as the basis for all subsequent processing steps. Evidently, the proposed network should satisfy the following requirements:

(1) Feature information contained in multilabel samples is more abundant and complex than that of single-label samples. Therefore, a feature extraction network needs to have sufficiently deep convolution layers and rich receptive fields.
(2) Considering that a defect inspection system needs to be quickly deployed and implemented in an actual production environment, the network requires efficient training and inference performance.

Relying on the above considerations and several CNN architectures reported in other research [16,17], we deployed Inception v3 [18] of GoogLeNet as a feature extraction network in FAR-Net. Inception v3 is an optimized version proposed by the Google team based on Inception v1. The advancement of this network mainly lies in the factorization of convolutions with a large filter size, the utility of auxiliary classifiers, and the efficient grid size reduction. Various research works, including experiments, have fully demonstrated its excellent performance in the field of image classification. Table 1 details the convolution layer parameters and the output size of each layer in Inception v3.

Let H denote an input defect sample, and $y={\left[y^{1},y^{2},\ldots ,y^{C}\right]}^{T}$ denote the ground truth label corresponding to the considered sample, where C is the number of labels in a dataset. Here, y can be expressed in one-hot form, meaning a binary indicator; $y^{l}=1$ denotes that the l-th label exists in the sample, l=1, 2, …, C; $y^{l}=0$ otherwise. Then, the feature extraction module used in FAR-Net can be described as follows:

(7) $$X=f_{Incep}\left(H,\theta_{Incep}\right),X\in R^{8\;\times \;8\;\times \;2048}$$

where X denotes the output feature map of the fully connected layer at the top of Inception v3 network.

### 2.2. Label-Wise Feature Aggregation

**Figure 5:**
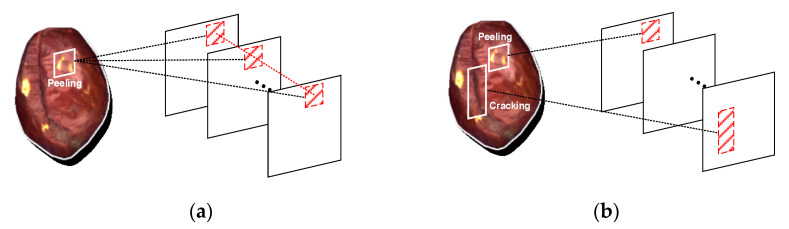
Correspondence between a defect sample and the feature map: (**a**) single-label situation; (**b**) multilabel situation.

The examples of feature maps extracted by CNN for an input sample are represented in Figure 5a. The information about the same region between different dimensions of a feature is related to the corresponding region of an input sample. Different dimensions focus on the diverse levels of a target region. However, concerning multilabel image classification, each dimension of a feature usually incorporates multiple defect features; therefore, it is obviously difficult to capture the semantic association between labels directly from these feature maps.

To enable better learning of correlation among different defects, we have attempted to aggregate label-wise features in the dimension of feature X, as depicted in Figure 5b. In this way, each dimension of a feature corresponds to a single defect, which is more convenient for subsequent modules to further learn semantic relations between labels.

**Figure 6:**
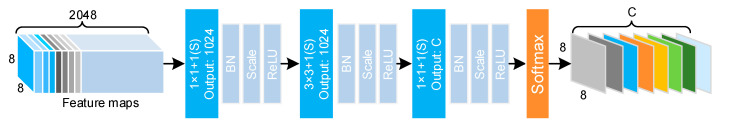
Structure of the label-wise feature aggregation module.

The structure of a label-wise feature aggregation module is represented in Figure 6. The feature map $X\in R^{8\;\times \;8\;\times \;2048}$ extracted by Inception v3 is used as the input into this module. To achieve a one-to-one correspondence between labels and feature channels, a convolutional block is employed to initially learn the conversion relationship between them:

(8) $$S=f_{seg}\left(X,\theta_{seg}\right),S\in R^{8\;\times \;8\;\times \;C}$$

Here, the convolutional block is implemented using three convolutional layers; the kernel size and the output number are 1 × 1 × 1024, 3 × 3 × 1024, and 1 × 1 × C, respectively. The output of each convolution layer corresponds to a batch normalization layer, a scale layer, and a ReLU activation layer. Here, $S\in R^{8\;\times \;8\;\times \;C}$ denotes the output feature of the 3rd convolutional block. In this case, we consider that each channel of S responds to a certain label in a dataset. Next, a Softmax layer is deployed to normalize each channel of S to obtain aggregated feature map A as follows:

(9) $$A^{l}\left(i,j\right)=\frac{exp\left(S^{l}\left(i,j\right)\right)}{\sum_{i,j}exp\left(S^{l}\left(i,j\right)\right)},i,j\;for\;all,A\in R^{8\;\times \;8\;\times \;C},l=1,\;2,\;\ldots ,\;C$$

where $S^{l}\left(i,j\right)\left(l=1,\;2,\;\ldots ,\;C\right)$ denotes the response value at the coordinate (i,j) of the l-th channel of feature S learned by the convolutional block, while $A^{l}\left(i,j\right)$ represents the response value at the (i,j) of the l-th channel of feature A after normalization.

However, in general, a given sample image does not contain all kinds of defects. Therefore, the channels of feature A corresponding to the labels that do not exist in an input image are usually useless, constituting so-called negative responses. On the contrary, the channels corresponding to the labels existing in an image correspond to positive responses. Obviously, negative responses are not helpful in the subsequent semantic relation learning and need to be deactivated, while positive ones require to be further activated.

### 2.3. Feature Activation and Deactivation

**Figure 7:**
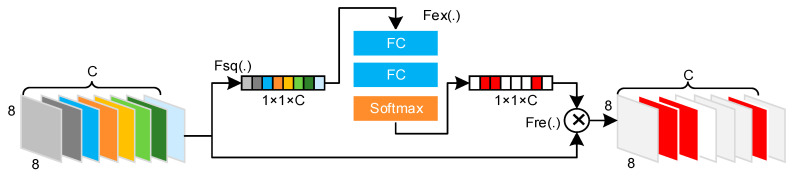
Structure of the activation and deactivation module.

To suppress the nonexistent label responses in feature A, a squeeze and excitation (SE) block inspired by the work of J. Hu et al. [19] is deployed to realize feature activation and deactivation, as shown in Figure 7.

It is generally considered that the importance of each channel in a feature maps is unequal in the current task. The SE block can be used to estimate the difference in the importance of these features through supervised learning. By weighting response values, redundant features can be deactivated, while valuable features are activated. The SE block is mainly divided into three steps: squeeze, excitation, and reweight. Squeeze aims to compress feature maps using global average pooling, converting each two-dimensional channel $A^{l}$ into real number $z_{l}$, which implies a global receptive field to a particular extent:

(10) $$z_{l}=F_{sq}\left(A^{l}\right)=\frac{1}{8\times 8}\sum_{j=1}^{8}\sum_{i=1}^{8}A^{l}\left(i,j\right),z_{l}\in R^{1\;\times \;1},l=1,\;2,\;\ldots ,\;C.$$

Excitation is realized to explicitly model the correlation among feature channels. It is implemented by two C-dimensional fully connected (FC) layers and one activation layer. Let $W_{1}$ and $W_{2}$ denote the learnable parameters of the first and second FC layers, respectively; then, the output feature of the 2nd FC layer can be expressed as follows:

(11) $$s_{l}=F_{ex}\left(z,W\right)=\sigma \left(g\left(z,W\right)\right)=\sigma \left(W_{2}\sigma \left(W_{1}z\right)\right),s_{l}\in R^{1\;\times \;1\;\times \;C},l=1,\;2,\;\ldots ,\;C.$$

Reweight is implemented to weight the output of excitation corresponding to feature A aiming to obtain $\tilde{A}\in R^{8\;\times \;8\;\times \;C}$:

(12) $${\tilde{A}}^{l}=F_{re}\left(A^{l},s_{l}\right)=A^{l}\cdot s_{l},l=1,\;2,\;\ldots ,\;C.$$

So far, the activated channel in feature $\tilde{A}$ corresponds to the existent label in an input image. The correlations between labels are directly manifested as those between channels, thereby enabling the subsequent relation learning.

### 2.4. Attention-Based Relation Learning

Most of the CNN-based classification algorithms do not exploit inherent connections between labels. Considering jujubes as an example, cracking tends to occur with rot or bird pecking, while russeting and shriveled symptoms may occur alone or with any other defect. Mining semantic relations between different defects may considerably improve the multilabel classification accuracy.

Inspired by A. Vaswani et al. [12] and H. Hu et al. [20], a multilabel-relation learning module is developed. The attention mechanism is implemented to fully understand the semantic relation between different labels. By integrating the label-wise independent features and correlation features, the model understanding of multilabel defects can be conspicuously improved.

**Figure 8:**
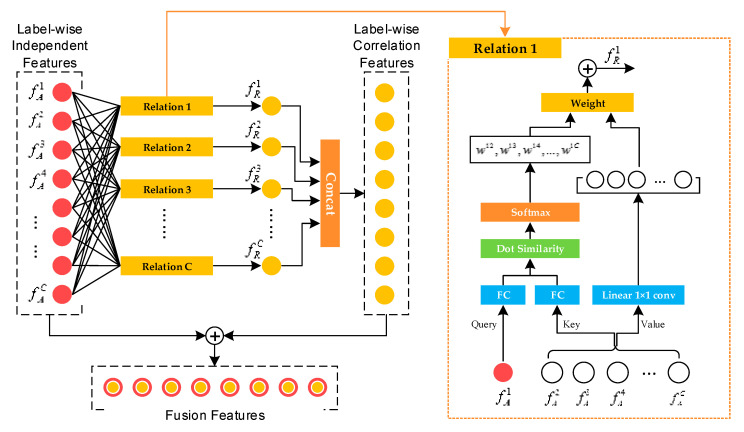
Structure of the attention-based relation learning module.

The structure of the ARL module is depicted in Figure 8. The input into this module is composed using the output of the LFA and ADA modules, which is denoted as $\left\{f_{A}^{l}\right\},l=1,\;2,\;\ldots ,\;C$. Then, C relation submodules are built, and the correlation features for each label are obtained, which is denoted as $\left\{f_{R}^{l}\right\},l=1,\;2,\;\ldots ,\;C$. Finally, the two feature maps are fused to obtain the final fusion feature for multilabel classification:

(13) $$\left\{f_{M}^{l}\right\}=\left\{f_{A}^{l}\right\}+\left\{f_{R}^{l}\right\},l=1,\;2,\;\ldots ,\;C$$

The upper part of Figure 8 illustrates the process of the submodule called relation. Intuitively, weights are always used to measure the degree of association between labels [21], which is consistent with the AM. Specifically, if there is a strong semantic correlation between a label and a query label, a larger weight is assigned to exert influence; otherwise, a smaller weight is set. This process can be expressed as followed:

(14) $$f_{R}^{l}=\overset{C}{\underset{m=1,m\neq l}{\sum }}w^{ml}\cdot \left(W_{V}\cdot f_{A}^{m}\right),l=1,\;2,\;\ldots ,\;C$$

where $f_{R}^{l}$ denotes the correlation feature of the l-th label that is obtained by the weighted addition of the label-wise independent features $\left\{f_{A}^{m}\right\},m=1,\;2,\;\ldots ,\;C$ (m≠l) after linear transformation $W_{V}$. The correlation weight $w^{ml}$ indicates the influence of the m-th label on the l-th one, which is obtained by the scaled dot-product attention algorithm [12]:

(15) $$w^{ml}=\frac{exp\left(w_{S}^{ml}\right)}{\sum_{k=1,k\neq l}^{C}exp\left(w_{S}^{kl}\right)},w_{S}^{ml}=\frac{W_{K}f_{A}^{m}\cdot W_{Q}f_{A}^{l}}{\sqrt{d_{K}}},l=1,\;2,\;\ldots ,\;C,m=1,\;2,\;\ldots ,\;C,m\neq l$$

where $w_{S}^{ml}$ denotes the semantic relevance between the m-th and l-th labels. In fact, the dot product is considered similar to the cosine distance in metric learning, which is deemed a reasonable method to measure the similarity of features. Here, $W_{K}$ and $W_{Q}$ are both linear transformations and they map features $f_{A}^{m}$ and $f_{A}^{l}$ to the same subspace to measure the similarity between them; $d_{K}$ is a hyperparameter, which was set to the baseline value of 64 [12] in this study.

Eventually, after the ARL, the label-wise fusion feature $\left\{f_{M}^{l}\right\}\in R^{8\;\times \;8\;\times \;C},l=1,\;2,\;\ldots ,\;C$ is obtained. Then, as shown in Figure 4, an 8 × 8 average pooling layer and a Sigmoid activation layer are used to obtain the multilabel classification result.

## 3. Experimental Evaluation and Module Discussion

### 3.1. Multilabel Jujube Defect Dataset

**Figure 9:**
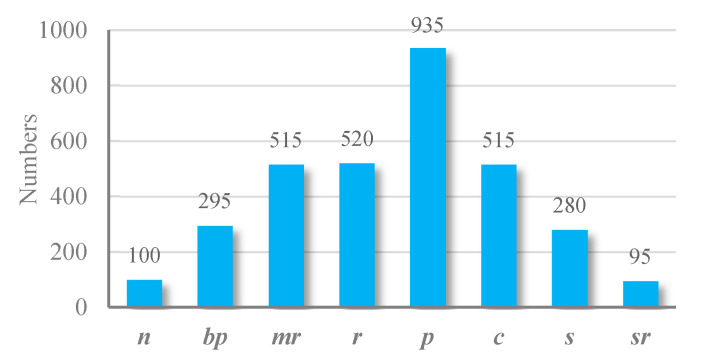
Label distribution in the multilabel jujube defect dataset.

A multilabel jujube defect dataset that was constructed for the purposes of the present study, comprised a total of eight labels: normal, russeting, mild rot, severe rot, cracking, shriveled, peeling, and bird pecking. The resulting dataset included 1930 samples. Among them, 660 samples were single label, 1200 were double label, and 70 were triple label. Multilabel samples accounted for 65.8% of the total. The dataset was divided into the training, verification, and test sets using the ratio of 3:1:1. The specific distributions of different samples and labels in the dataset are listed in Table 2 and represented in Figure 9. In the data preprocessing stage, all samples were resized to 299 × 299 to meet the input requirement of Inception v3. Then, the semi-supervised data augmentation method (SSDA) [22] was used for data augmentation.

### 3.2. Model Training

The specific configurations of the experimental platform are listed in Table 3. In the present study, FAR-Net was implemented and trained using the Python interface deployed in Caffe [23]. Hyperparameters used to train the network are listed in Table 4.

The training of the entire FAR-Net model was divided into four stages:

(1) The FE module was fine-tuned on the multilabel jujube defect dataset, while the initial parameters of the model were obtained by pretraining on the ImageNet single-label dataset.
(2) The parameters of the FE module were fixed, and the LFA and ADA modules were trained.
(3) The parameters of the first three modules were fixed, and the ARL module was trained.
(4) The overall model was fine-tuned simultaneously on the multilabel dataset.

**Figure 10:**
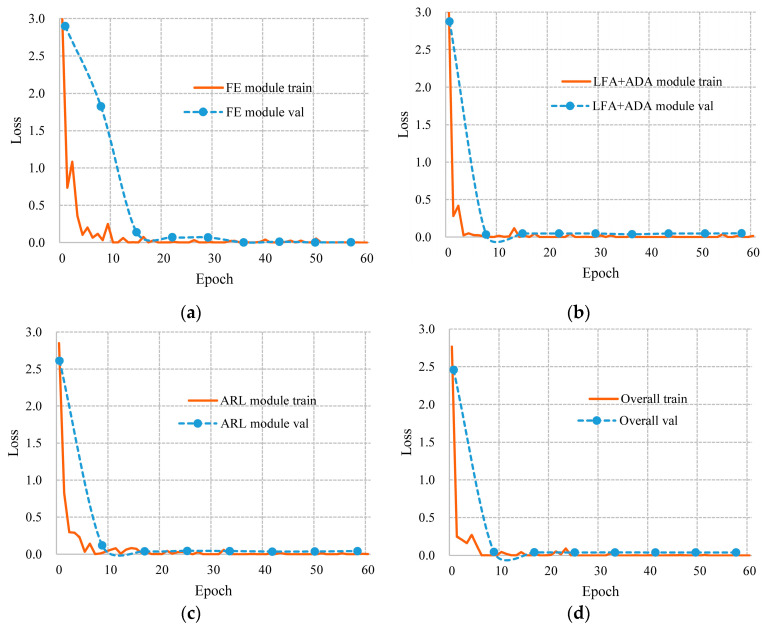
Training baselines of the four training stages in FAR-Net: (**a**) FE module fine-tuning; (**b**) LFA and ADA module training; (**c**) ARL module training; (**d**) overall model fine-tuning.

The cross-entropy loss function was used during the whole training process as follows:

(16) $$L_{loss}\left(y,\hat{y}\right)=\sum_{l=1}^{C}y_{l}\log\sigma \left({\hat{y}}_{l}\right)+\left(1-y_{l}\right)\log\left(1-\sigma \left({\hat{y}}_{l}\right)\right)$$

where y and $\hat{y}$ denote the ground truth label and the predicted label of an input sample, respectively. The training baselines for each of the above stages are depicted in Figure 10. It was confirmed that the module-wise training strategy could effectively accelerate and ensure the convergence of the whole model.

### 3.3. Experimental Results

Discussions presented in Section I.B imply that, at present, multilabel classification based on deep learning mainly includes two types of approaches: CNN-based and RNN-based methods. The latter, including LSTM, tend to have low calculation efficiency and a large need for memory. This is not suitable for rapid deployment in actual production. Therefore, we focused on several typical and state-of-art CNN networks (including AlexNet [24], VGG-16 [25], and Inception v3) and utilized them as benchmark approaches in an experiment. All of the above networks were initialized from ImageNet-trained weights. Here, Inception v3 could be regarded as an analog of the proposed FAR-Net model but without a label-relation learning mechanism. Therefore, we performed the comparison between them to precisely evaluate the performance of this mechanism that was the core module of FAR-Net.

**Figure 11:**
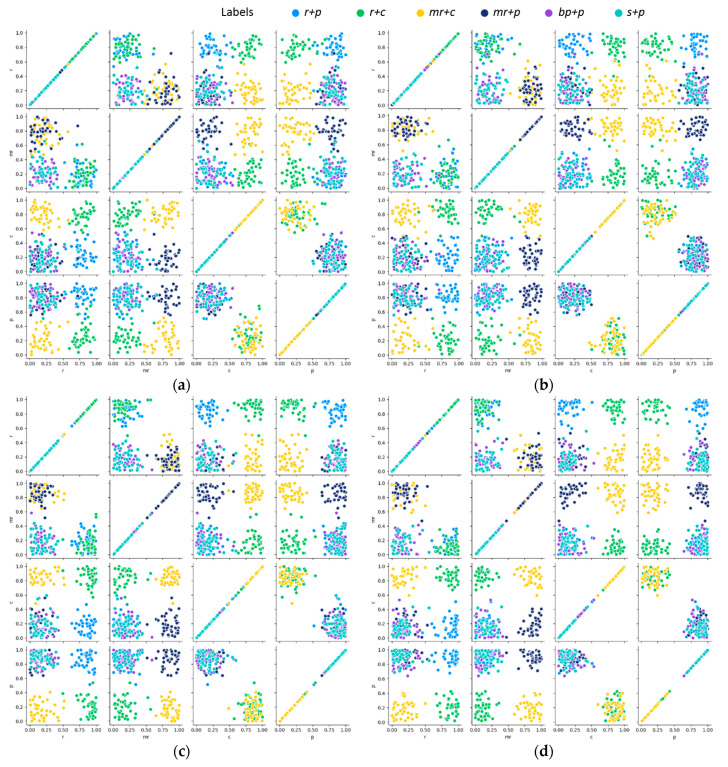
Label-wise prediction confidence grid on the multilabel jujube defect dataset for different models: (**a**) AlexNet; (**b**) VGG-16; (**c**) Inception v3; (**d**) FAR-Net.

To intuitively represent the discrimination of multilabel samples on different models, we innovatively introduced a label-wise prediction confidence grid to show the distribution of samples, as shown in Figure 11. The prediction result of a sample can be expressed as ${\hat{y}}_{P}=\left[{\hat{y}}_{P}^{1},{\hat{y}}_{P}^{2},\ldots ,{\hat{y}}_{P}^{C}\right]$ after the last Sigmoid layer. ${\hat{y}}_{P}^{l}\in \left[0,1\right]$ denotes the confidence of each label (l=1,2,…,C). For the convenience of graphing, four labels, namely *r*, *mr*, *c*, and *p*, with the highest frequency in the jujube defect dataset were selected. Then the prediction results of all samples can be expressed as:

(17) $${\hat{Y}}_{P}={\left[{\hat{y}}_{P1},{\hat{y}}_{P2},\ldots ,{\hat{y}}_{PN}\right]}^{T},{\hat{y}}_{Pk}=\left[{\hat{y}}_{P}^{r},{\hat{y}}_{P}^{mr},{\hat{y}}_{P}^{c},{\hat{y}}_{P}^{p}\right],k=1,2,\ldots ,N$$

where N denotes the number of samples in the test set. Each label confidence in ${\hat{Y}}_{P}$ was mapped onto a column and row in a grid of multiple axes to reveal the pairwise relationship between different labels. Generally, when concerning an ideal classifier, the confidence of labels that exist in the sample and labels that do not exist will vary considerably. It can be inferred from Figure 11 that FAR-Net represents preferable discrimination of confidence value, which indicated a better classification performance for multilabel images.

To further quantitatively evaluate the performance of different models, several indicators were selected, including average precision (AP), mean of AP (mAP), micro-F1, and macro-F1, which were used in [26]. The criteria of precision and recall were employed as follows:

(18) $$precision=\frac{TP}{TP+FP},recall=\frac{TP}{TP+FN}$$

where TP and FN denote the ratios of defective samples detected as defective and nondefective, respectively, while FP denotes the ratio of nondefect samples falsely detected as defective.

**Figure 12:**
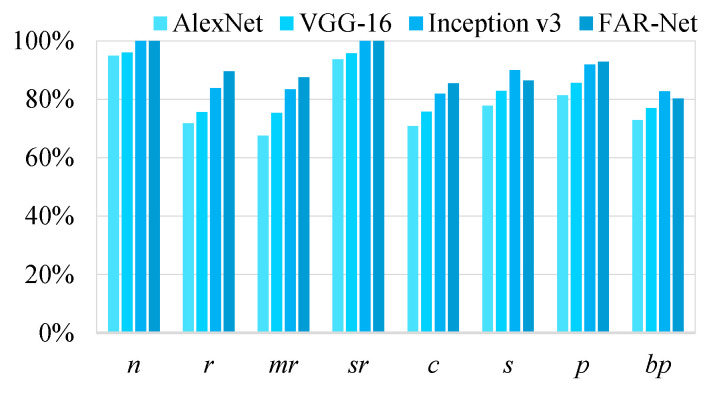
Recall of the four networks on the jujube defect dataset.

**Figure 13:**
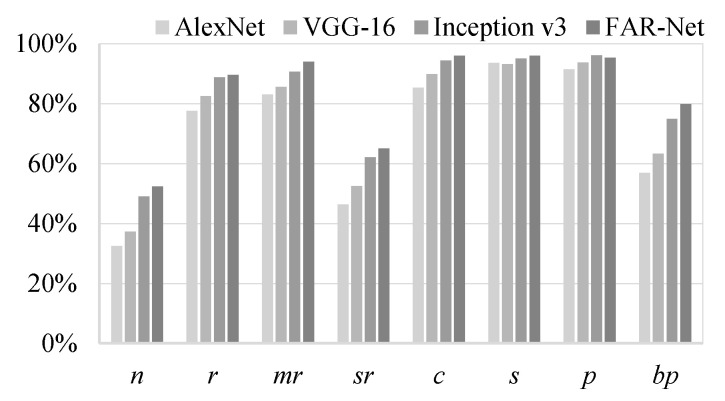
Precision of the four networks on the jujube defect dataset.

The recall and precision of the four models on the multilabel jujube defect dataset are depicted in Figure 12 and Figure 13, respectively. The APs of different labels are provided in Table 5. Notably, FAR-Net achieved better results for six labels out of a total of eight, compared with the other considered methods, and its mAP (mean average precision) was the best, reaching 90.28%, which was higher than 89.25% of Inception v3, 87.18% of CNN-RNN, 82.99% of VGG-16, and 78.87% of AlexNet. Specifically, all four considered models demonstrated relatively high precision for the two labels: *normal* and *severe rot*. The reason was that although these two labels occurred less frequently in the dataset, they almost did not appear conjointly with other labels, which meant the approximation to the single-label classification problem and accordingly, the accuracy was relatively higher. In contrast, the label called *peeling* often occurred together with several other defects, which made it difficult to discriminate. However, as it had the highest frequency in the dataset (48.4% of the total samples), it provided the model with more opportunities to learn; therefore, the classification result was satisfactory. The other labels that tended to occur with *peeling* were *russeting*, *mild rot*, and *cracking*. Owing to the relation learning mechanism, the AP value of FAR-Net increased by 5.77%, 4.07%, and 3.50%, respectively, compared to Inception v3, which was significantly improved as well.

In addition, the micro-F1 and macro-F1 scores [14] for different models are listed in Table 6. F1 scores are balanced metrics considering precision and recall simultaneously. It can be inferred that FAR-Net achieved satisfactory classification performance with an acceptable testing time. The observed results indicated that the proposed method could comprehensively learn the correlations between different labels and improved the multilabel classification results.

### 3.4. Module Discussion

To further explore the performance of four modules in FAR-Net and analyze the contribution of each module to the improvement of multilabel classification accuracy, a module-wise occlusion experiment was conducted. The results are presented in Table 7.

(1) FAR-Net was equivalent to Inception v3 after removing LFA, ADA, and ARL modules. In this case, mAP was 89.25% on the multilabel jujube dataset.
(2) The value of mAP reached 89.40% when the LFA and ADA modules were added, which was only 0.15% higher than that of Inception v3. This indicated that adding label feature separation only did not result in a considerable improvement in the overall classification outcome. This was because the model only extracted the label-wise independent features and did not learn the semantic correlation between labels.
(3) The value of mAP achieved 90.28% when the ARL module was also added, which was 0.88% higher than that in step (2).
(4) The value of mAP achieved only 89.85% when ADA module was removed, which indicated that label-wise feature aggregation is not enough for relation learning because negative responses need to be deactivated, while positive ones need to be further activated.

The module-wise occlusion experiment results indicated that the ARL module could effectively learn internal connections between labels and therefore improved the classification performance.

## 4. Conclusions

Multilabel image classification is always a hot issue in multimedia processing. This is not only because it is more challenging than single-label classification, but also closer to real-world situations. In this study, we introduced a feature-wise attention-based relation network. The proposed network model was capable of learning correlation and dependencies between different labels owing to four different modules: feature extraction, label-wise feature aggregation, activation and deactivation, and attention-based relation learning module. Experimental results on a multilabel jujube defect dataset indicated that FAR-Net had significant advancement and effectiveness in the classification of multilabel defects. Overall, via a CNN-attention architecture, this approach provides a clear path toward higher precision and stronger robustness for the traditional industry of agricultural product sorting.

In the future, we will further investigate the labeling of multilabel defects through a semi-supervised or unsupervised way. As in real production, factories often need to update or iterate the surface defect inspection system in the short term. We will combine the inference ability of existing models and the prior knowledge of experts to improve the positioning efficiency of multilabel defects in our future work.

## Figures and Tables

**Table 1:** Convolutional layer parameters and feature size of Inception V3.

**Phrase**	**Conv1**	**Conv2_x**	**Conv3_x**	
**Feature size**	149 × 149 × 32	147 × 147 × 64	71 × 71 × 192	
**Conv params**	3×3.32	3×3.32 3×3.64	1×1.80 3×3.192	
**Phrase**	**Conv4_x**	**Conv5_x**	**Conv6_x**	**Linear**
**Feature size**	35 × 35 × 288	17 × 17 × 768	8 × 8 × 2048	1 × 1
**Conv params**	$\left[\begin{array}{c} 1\times 1.208\\ 3\times 3.192\\ 5\times 5.64 \end{array}\right]\times 1$ $\left[\begin{array}{c} 1\times 1.240\\ 3\times 3.192\\ 5\times 5.64 \end{array}\right]\times 2$	$\left[\begin{array}{c} 1\times 1.640\\ 1\times 7.448\\ 7\times 1.448 \end{array}\right]\times 1$ $\left[\begin{array}{c} 1\times 1.704\\ 1\times 7.512\\ 7\times 1.512 \end{array}\right]\times 2$ $\left[\begin{array}{c} 1\times 1.768\\ 1\times 7.576\\ 7\times 1.576 \end{array}\right]\times 1$	$\left[\begin{array}{c} 1\times 1.1344\\ 1\times 3.768\\ 3\times 1.768\\ 3\times 3.384 \end{array}\right]\times 2$	Avg pooling C-d FC Sigmoid

**Table 2:** Samples distribution in the multilabel jujube defect dataset.

Sample	No.	Sample	No.	Sample	No.
*n* ^1^	100	*r + p*	200	*r + p + mr*	20
*r*	80	*r + c*	200	*r + p + c*	20
*mr*	80	*mr + p*	200	*mr + c + p*	15
*sr*	80	*mr + c*	200	*bp + sr + c*	15
*c*	80	*bp + p*	200		
*s*	80	*s + p*	200		
*p*	80				
*bp*	80			Total	1930

^1^*n*: normal, *r*: russeting, *mr*: mild rot, *sr*: severe rot, *c*: cracking, *s*: shriveled, *p*: peeling, *bp*: bird pecking.

**Table 3:** Configuration of the experimental platform.

CPU:	Intel E3-1230 V2*2 (3.30 GHz)
Memory:	16 GB DDR3
GPU:	NVIDIA Tesla K20
OS:	Ubuntu 16.04 LTS
Compiler:	Visual Studio Code with Python 2.7

**Table 4:** Hyperparameter settings in the FAR-Net training.

Momentum:	0.9
Weight decay:	0.0005
Base learning rate:	0.001
Learning rate policy:	Exponential
Batch size:	16

**Table 5:** Average precision of the four networks on the jujube defect dataset.

	*n*	*r*	*mr*	*sr*	*c*	*s*	*p*	*bp*	mAP
AlexNet	95.00%	71.73%	67.57%	93.68%	70.87%	77.86%	81.39%	72.88%	78.87%
VGG-16	96.00%	75.58%	75.34%	95.79%	75.73%	82.86%	85.67%	76.95%	82.99%
CNN-RNN	**100.00%**	82.47%	81.95%	**100.00%**	78.86%	87.35%	88.08%	78.74%	87.18%
Inception v3	**100.00%**	83.85%	83.50%	100.00%	81.94%	**90.00%**	91.98%	**82.71%**	89.25%
**FAR-Net**	**100.00%**	**89.62%**	87.57%	**100.00%**	**85.44%**	86.43%	**92.83%**	80.34%	**90.28%**

**Table 6:** Other experimental results on the jujube defect dataset for different models.

Network	Micro-F1	Macro-F1	Testing Time (s)
AlexNet	74.64%	71.50%	0.67
VGG-16	78.67%	76.21%	0.81
CNN-RNN	83.04%	81.61%	0.93
Inception v3	85.13%	83.55%	0.55
FAR-Net (without ARL)	85.35%	84.01%	0.61
**FAR-Net**	**86.77%**	**85.42%**	0.88

**Table 7:** Classification results of the module-wise occlusion experiment.

Network	mAP
FAR-Net (without LFA, ADA, and ARL)	89.25%
FAR-Net (without ARL)	89.40%
FAR-Net (without ADA)	89.85%
FAR-Net	90.28%

## Data Availability

Data sharing not applicable.
